# Supplementary material for: Herpes simplex virus infection, Acyclovir and IVIG treatment all independently cause gut dysbiosis
Source: PLoS One. 2020 Aug 6;15(8):e0237189. doi: 10.1371/journal.pone.0237189 (PMC7410316; doi:10.1371/journal.pone.0237189)
Supplement: S1 Fig — For each taxa, edgeR analyses determined that the only pairwise difference was between ACV treated and untreated uninfected female mice (FDR-adjusted P values < 0.05). Bars = standard error. Females = _F and Males = _M. (PDF) [file pone.0237189.s002.pdf]

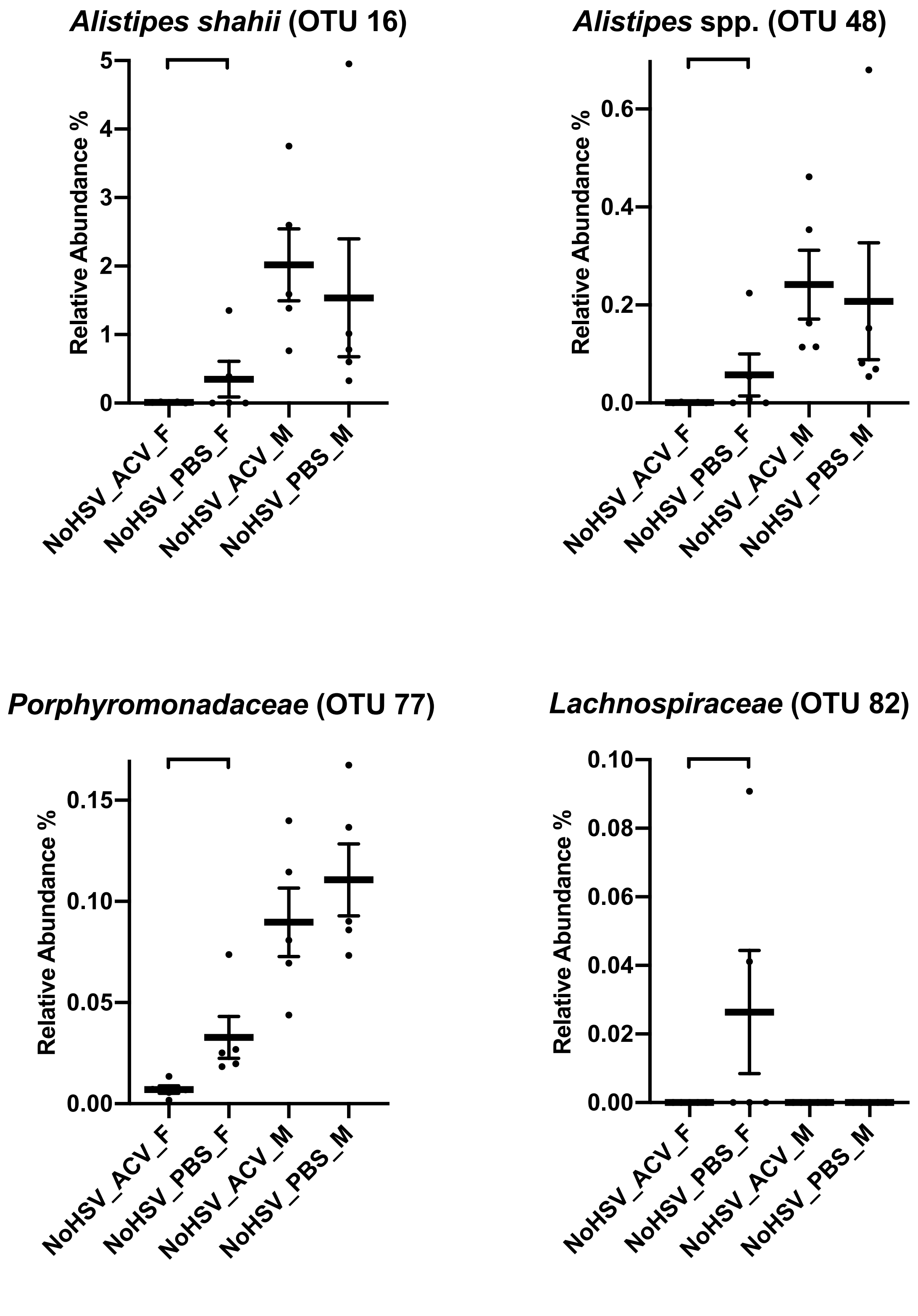

Supplemental Figure 1. Fecal Bacteria from Uninfected Mice Treated and Not Treated with ACV. For each taxa, edgeR analyses determined that the only pairwise difference was between ACV treated and untreated uninfected female mice (FDR-adjusted P values < 0.05). Bars = standard error. Females = _F and Males = _M.
