# Supplementary material for: Herpes simplex virus infection, Acyclovir and IVIG treatment all independently cause gut dysbiosis
Source: PLoS One. 2020 Aug 6;15(8):e0237189. doi: 10.1371/journal.pone.0237189 (PMC7410316; doi:10.1371/journal.pone.0237189)
Supplement: S2 Fig — Principal-coordinates analysis (PCoA) of Hellinger beta diversity distance values generated from 16S rRNA gene sequences. The number of mice (n) in each genotype-microbiota group are shown in parentheses. Females = _F and Males = _M. (PDF) [file pone.0237189.s003.pdf]

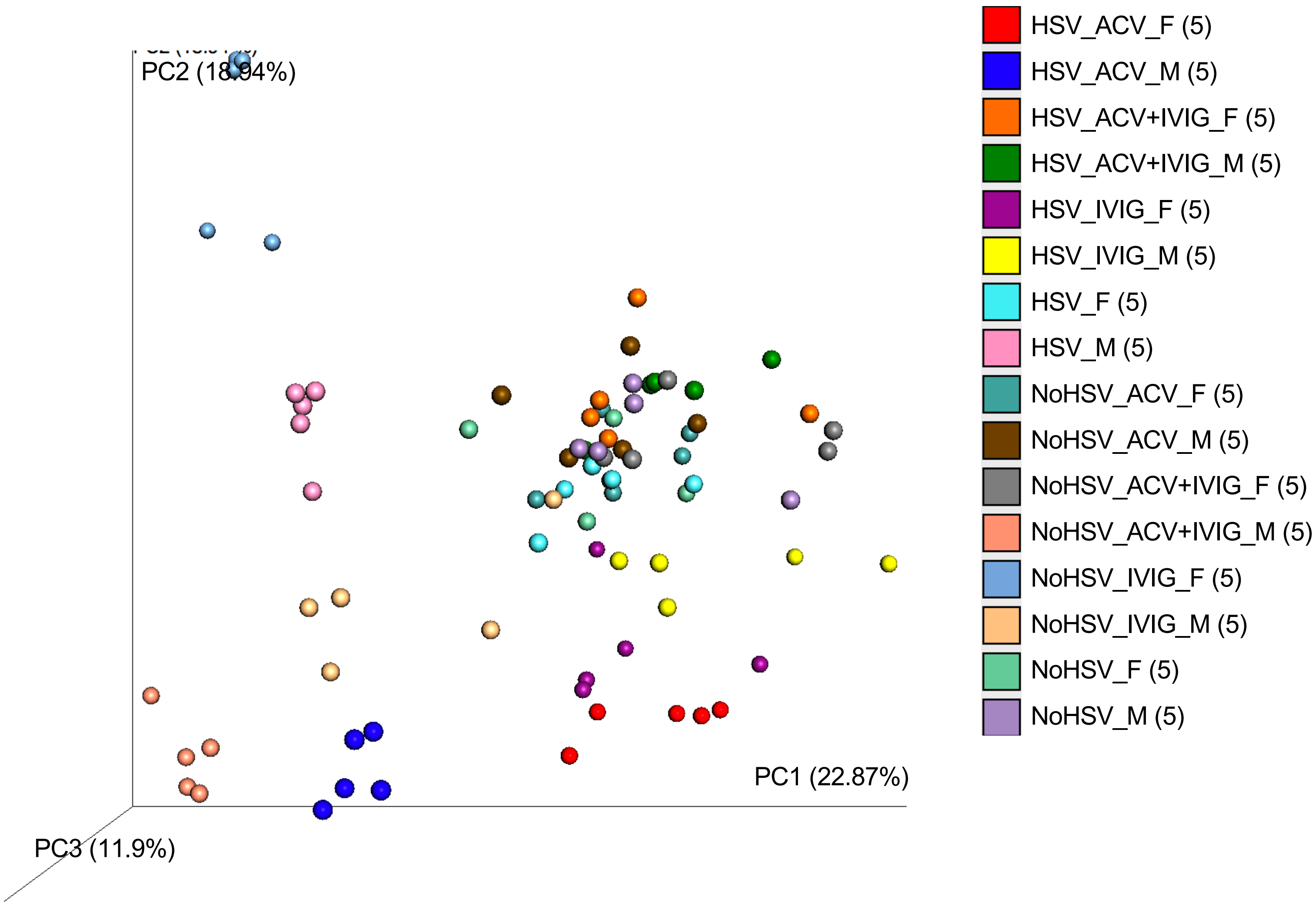

Supplemental Figure 2. Beta Diversity Analysis of Fecal Bacteria from HSV-Infected and Uninfected Mice Treated and Not Treated with ACV and/or IVIG. Principal-coordinates analysis (PCoA) of Hellinger beta diversity distance values generated from 16S rRNA gene sequences. The number of mice (n) in each genotype-microbiota group are shown in parentheses. Females = _F and Males = _M.
