# Supplementary material for: Herpes simplex virus infection, Acyclovir and IVIG treatment all independently cause gut dysbiosis
Source: PLoS One. 2020 Aug 6;15(8):e0237189. doi: 10.1371/journal.pone.0237189 (PMC7410316; doi:10.1371/journal.pone.0237189)
Supplement: S4 Fig — Pairwise differences are shown by horizontal lines (edgeR, FDR-adjusted P values < 0.05). Bars = standard error. Females = _F and Males = _M. (PDF) [file pone.0237189.s005.pdf]

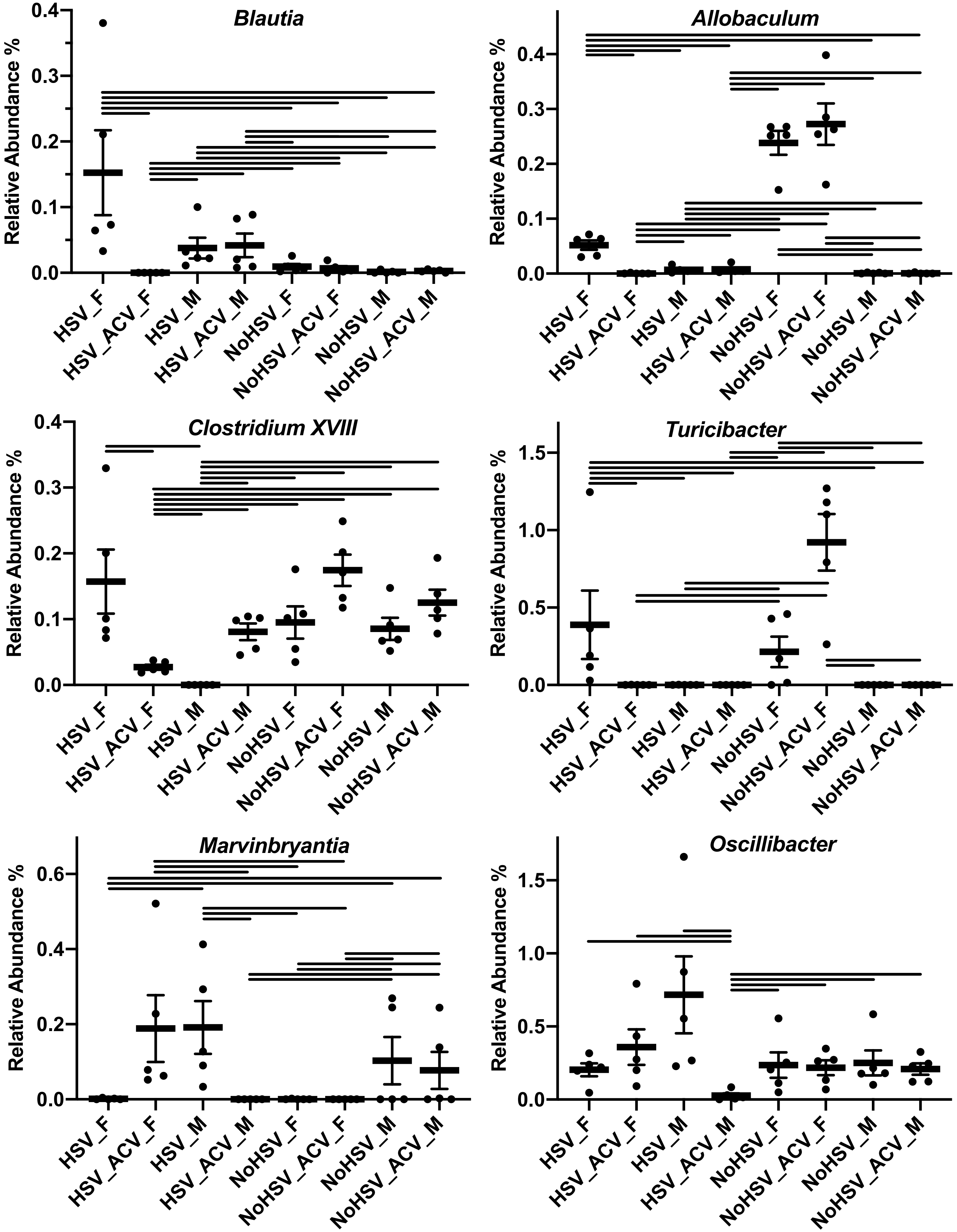

S4 Fig. Fecal Bacterial Genera from HSV-Infected and Uninfected Mice Treated and Not Treated with ACV and/or IVIG. Pairwise differences are shown by horizontal lines (edgeR, FDR-adjusted P values < 0.05). Bars = standard error. Females = _F and Males = _M.
