# Supplementary material for: Herpes simplex virus infection, Acyclovir and IVIG treatment all independently cause gut dysbiosis
Source: PLoS One. 2020 Aug 6;15(8):e0237189. doi: 10.1371/journal.pone.0237189 (PMC7410316; doi:10.1371/journal.pone.0237189)
Supplement: S1 Table — (PDF) [file pone.0237189.s006.pdf]

adonis
hellinger
Supplementary Table. The numbers in the cells are P values obtained from Adonis tests of the Hellinger beta diversity matrix.

	HSV_ACV_F	HSV_ACV_M	HSV_ACVplusIVIG_F	HSV_ACVplusIVIG_M	HSV_IVIG_F	HSV_IVIG_M	HSV_PBS_F	HSV_PBS_M
HSV_ACV_F								
HSV_ACV_M	0.009							
HSV_ACVplusIVIG_F	0.01	0.009						
HSV_ACVplusIVIG_M	0.006	0.012	0.02					
HSV_IVIG_F	0.028	0.007	0.009	0.005				
HSV_IVIG_M	0.005	0.011	0.004	0.011	0.004			
HSV_PBS_F	0.01	0.015	0.011	0.01	0.012	0.009		
HSV_PBS_M	0.013	0.008	0.004	0.008	0.015	0.013	0.01	

	NoHSV_ACV_F	NoHSV_ACV_M	NoHSV_ACVplusIVIG_	NoHSV_ACVplusIVIG_M	NoHSV_IVIG_F	NoHSV_IVIG_M	NoHSV_PBS_F	NoHSV_PBS_M
NoHSV_ACV_F								
NoHSV_ACV_M	0.015							
NoHSV_ACVplusIVIG_F	0.013	0.083						
NoHSV_ACVplusIVIG_M	0.009	0.012	0.004					
NoHSV_IVIG_F	0.008	0.005	0.014	0.011				
NoHSV_IVIG_M	0.016	0.008	0.01	0.012	0.007			
NoHSV_PBS_F	0.33	0.01	0.007	0.008	0.009	0.012		
NoHSV_PBS_M	0.01	0.712	0.036	0.002	0.007	0.01	0.006	
